# Langerhans cell histiocytosis in adults is associated with a high prevalence of hematologic and solid malignancies

**DOI:** 10.1002/cam4.1844

**Published:** 2018-12-30

**Authors:** Jennifer Ma, James H. Laird, Karen W. Chau, Monica R. Chelius, Benjamin H. Lok, Joachim Yahalom

**Affiliations:** ^1^ Department of Radiation Oncology Memorial Sloan Kettering Cancer Center New York New York; ^2^ Albert Einstein College of Medicine Bronx New York; ^3^ New York University School of Medicine New York New York; ^4^ Geisel School of Medicine Dartmouth College Hanover New Hampshire; ^5^ Radiation Medicine Program Princess Margaret Cancer Centre Toronto Ontario Canada

**Keywords:** eosinophilic granuloma, histiocytosis, Langerhans, Langerhans cell histiocytosis, second malignancies

## Abstract

**Background:**

Langerhans cell histiocytosis (LCH) is a rare disorder of histiocyte proliferation. Previous case studies suggest a higher prevalence of hematologic and solid malignancies among LCH patients, possibly due to treatment with tumorigenic agents such as etoposide. We report the first large, single‐institution experience of adult LCH patients with additional malignancies to study the characteristics of these patients.

**Methods:**

We identified 132 consecutive patients >18 years of age with histologically confirmed LCH at our center between 1990 and 2015. Demographics and detailed oncologic history were recorded to identify patients with additional malignancies.

**Results:**

Of 132 adult LCH patients, 42 (32%) patients had an additional malignancy. There were 53 malignancies among the 42 patients, with 31 (58%) preceding LCH diagnosis, 11 concurrent (≤3 months; 21%) with LCH diagnosis, and 11 (21%) after. Median age was 54 years (range 28‐89) with a median follow‐up of 3.7 years (0.1‐22.2) for this cohort. OS at 3 years was 98% in patients with LCH alone and 82% among patients with additional malignancies, with 30 (71%) alive at last follow‐up. Solid tumors, lymphomas, and other hematologic malignancies were observed as follows: 39 (74%), 9 (17%), and 5 (9%).

**Conclusion:**

Our cohort of adult LCH patients demonstrates an unusually high number of additional malignancies. Our study includes predominantly malignancies diagnosed preceding or concurrent with LCH, suggesting a cause of malignancy independent of LCH treatment. Further exploration of the biology of this rare disease may elucidate the mechanism of frequent additional malignancies.

## 1 INTRODUCTION

Langerhans cell histiocytosis (LCH) is a rare disorder of histiocyte proliferation with a highly varied clinical presentation, affecting single or multiple organ systems.^1^ It occurs predominantly in children and is less commonly seen in adults, with an incidence of 1‐2 cases per million adults.^2^ Therefore, the biology and therapeutics of adult LCH development and treatment are not well understood. Although there have been several large‐scale studies evaluating appropriate treatment for LCH in children, there is no current standard of care in adults.^3^, ^4^, ^5^, ^6^

Single‐system disease is more commonly observed in older children and adults, while acute multisystem disease is associated with children <3 years old.^1^ The most commonly affected organ systems are skeleton (77%) and skin (39%), but the lung, lymph nodes, liver, spleen, oral mucosa, and central nervous system are known to be affected by LCH as well.^7^ Pulmonary LCH, also referred to as eosinophilic granuloma of the lung, is predominantly seen in adults and is strongly associated with cigarette smoking. Despite the varied clinical presentation of LCH, the histological presentation is consistent with diagnosis by positive immunohistochemical staining for S100 with CD1a and/or CD207.^9^

The category of LCH has long been debated as either a neoplasm or a reactive condition resulting from immunologic dysfunction. Prior observations of increased cytokines and growth factors, and regulatory T‐cell expansion in LCH patients, suggested a systemic inflammatory response.^10^ However, the recent discovery of mutations in the *BRAF V600E* or *MAP2K1* oncogenes in over half of LCH patients, as well as clonality of the disease, strongly suggests that LCH is a neoplasm.^11^, ^12^, ^13^ This has significant implications on our understanding of LCH and its development.

The increased rate of additional malignancies in LCH patients has been observed in early literature reports published over 2‐3 decades ago.^14^, ^15^, ^16^ Notably, the literature has included mixed case reports of adults and children, or small case series focused on the pediatric LCH population. However, to our knowledge, there has not been a large study of other malignancies in consecutive adult LCH patients. Here, we report on our 25‐year single‐institution experience of additional malignancies in adult LCH patients, for whom the disease process and treatment options are poorly understood.

## 2 MATERIALS AND METHODS

### 2.1 Data collection

This retrospective study was independently reviewed and approved by the Institutional Review Board. We identified 132 consecutive patients >18 years of age who presented with histologically confirmed LCH (S100+, CD1a+) at our center between 1990 and 2015. Demographic information, disease characteristics, treatment, outcomes, and detailed oncologic history were recorded to identify patients with additional malignancies, excluding non‐melanoma skin cancers. LCH was classified by single or multisystem involvement and risk‐organ involvement. Risk‐organs for adult LCH were defined as the liver, spleen, and hematopoietic and central nervous systems, as recommended by Euro‐Histo‐Net's expert panel.^17^ Additional malignancies were classified as diagnosed preceding, concurrent with (within 3 months), or after LCH diagnosis. Patients with a concurrent pulmonary LCH and lung cancer were not included in the study as a localized reactive histiocytosis could not be excluded.

### 2.2 Statistical analysis

The Fisher's exact test was used for categorical variables, and the Wilcoxon rank sum test was used for continuous variables. The Kaplan‐Meier method was used to estimate overall survival, which was calculated from date of diagnosis until date of death or last follow‐up.

An age‐matched population analysis was performed using the incidence of cancer by age as reported by the American Cancer Society in 2017.^18^ The overall incidence by age was determined from these data by averaging the incidence of men and women.

## 3 RESULTS

### 3.1 Overall incidence of additional malignancies

The incidence of additional malignancies among our LCH cohort is significantly elevated as compared to the national incidence among the US population in 2017^18^ (Table 1) in all age groups. The largest differences are noted in the younger age groups, with incidence of additional malignancy in our cohort at ages 0‐49, 50‐59, 60‐69, and >70 at 17.4%, 17.6%, 25%, and 30.1% respectively. The incidence of cancer in an age‐matched US population in 2017 was 4.4%, 6.15%, 12%, and 29.6%, respectively.

**Table 1:** Age‐matched population analysis of cancer incidence among our cohort compared with the US population^18^

Age	Our cohort (%)	US population (%)	N
0‐49	17.4	4.4	132
50‐59	17.6	6.2	68
60‐69	25.0	12.0	40
70+	30.1	29.6	13

### 3.2 Additional malignancies cohort

**Figure 1:**
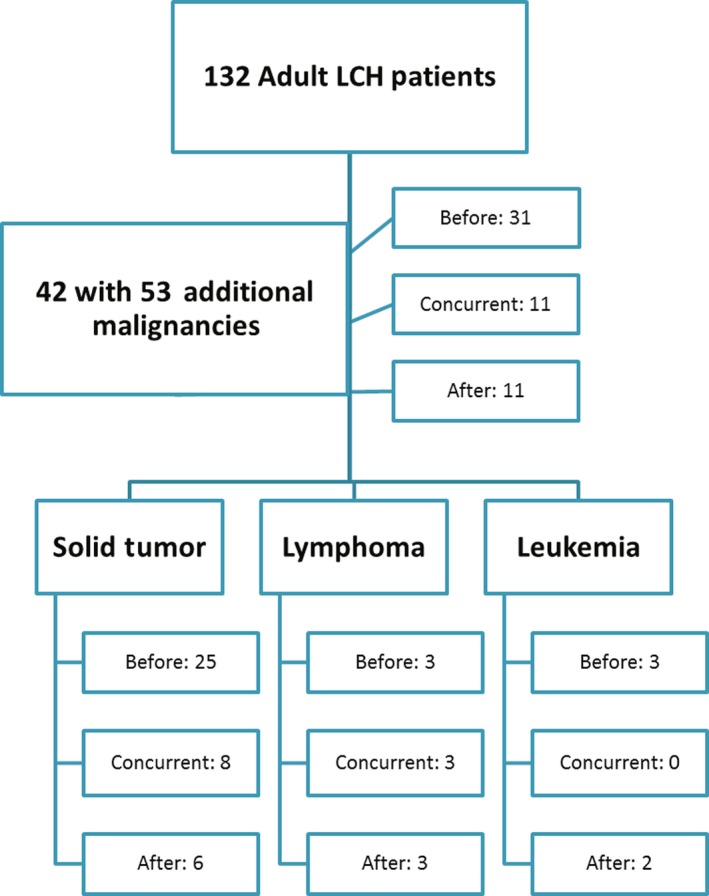
Cohort selection and distribution of additional malignancies in adult Langerhans cell histiocytosis patients (LCH)

**Figure 2:**
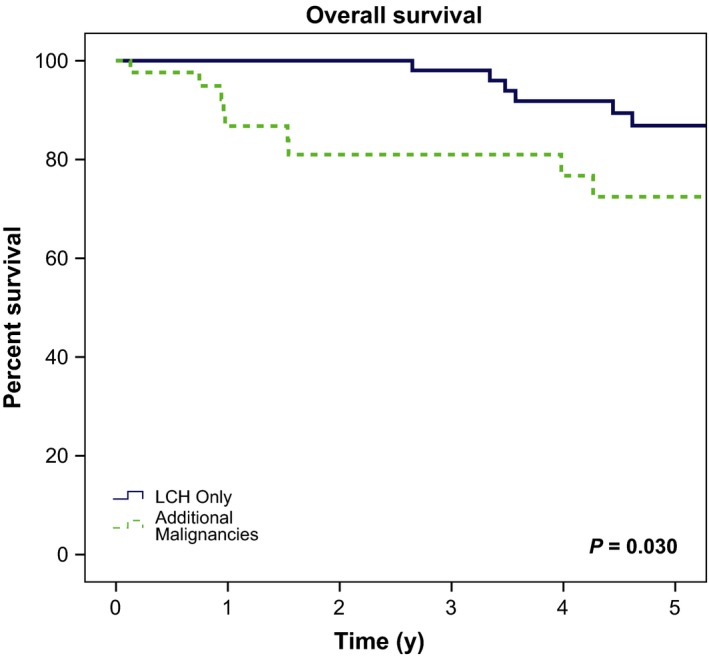
Kaplan‐Meier overall survival outcomes for the Langerhans cell histiocytosis‐only and additional malignancy cohorts

**Figure 3:**
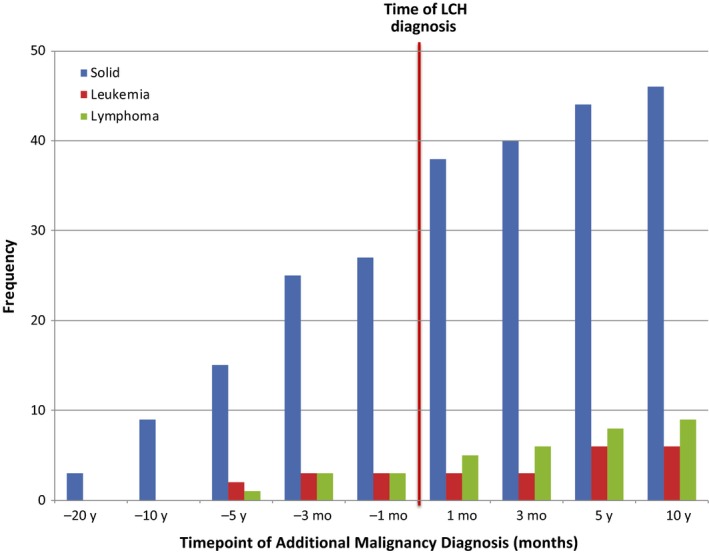
Cumulative number of additional malignancies over time, relative to date of Langerhans cell histiocytosis diagnosis

**Figure 4:**
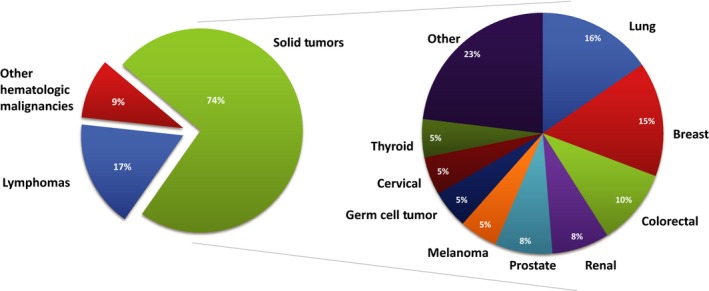
Distribution of additional malignancies by cancer type and histology

Of 132 consecutive adult LCH patients, 42 (32%) patients had an additional malignancy (Figure 1). Of the cohort of patients with additional malignancies, median age was 54 years (range 28‐89) with a median follow‐up of 3.7 years (0.1‐22.2; Table 2). Overall survival at 3 years was 82% (Figure 2), with 30 (71%) alive at last follow‐up. Active non‐LCH malignancy was the cause of death in 9 (75%) of deceased patients with cause of death unknown for the remaining three. The most common site of LCH involvement was lung in 25 patients (60%). Less common sites of involvement were lymph nodes (six patients; 14%), bone and skin (five patients each; 12%). Thirty patients (71%) were smokers, with an average smoking history of 35 pack‐years (range 0.6‐150). Thirty‐eight patients (90%) had single‐system LCH disease. There was a total of 53 non‐LCH malignancies among the 42 patients, with 31 (58%) preceding LCH diagnosis, nine concurrent (≤3 months; 21%) with LCH diagnosis, and 9 (21%) after (Table 3, Figure 3). Ten patients presented with two malignancies in addition to their LCH diagnosis, and two patients presented with ≥3 malignancies. There were 39 solid tumors (74%), nine lymphomas (17%), and five other hematologic malignancies (9%; Figure 4). Of the 42 patients with additional malignancies, six patients received systemic treatment for their LCH disease (three with multisystem disease, three with single‐system disease), four underwent resection, and the remainder underwent observation. None of the patients in this cohort received etoposide as part of their LCH treatment.

**Table 2:** Cohort demographics and Langerhans cell histiocytosis (LCH) disease characteristics

Characteristic	Additional malignancy cohort			LCH only			*P*‐value
	n = 42	%	Range	n = 90	%	Range	
Age	54		28‐89	42		18‐87	
Single system	38	90		69	77		0.038
Risk organ involvement	2	5		18	20		
Sites of LCH							
Lung	25	60		15	17		
Bone	5	12		48	53		
Lymph Nodes	6	14		8	9		
Skin	5	12		21	23		
Mucosa	1	2		4	4		
Brain	2	5		14	16		
Kidney	1	2					
Colon	1	2					
Liver				3	3		
Spleen				1	1		
Thyroid	1	2		1	1		
Parotid				1	1		
Blood							
Smokers	30	71		49	54		0.033
Average pack‐year	35		0.6‐150	33.1		0.2‐80	
3‐y OS		82			98		
Alive at last follow‐up	30	71		80	89		0.008
Median follow‐up (years)	3.7		0.1‐22.2	3.7		0.04‐20.1	

**Table 3:** Comparison of malignancy type and timing between our cohort and a large survey‐based study based on number of patients in our study (n = 42) and those of Egeler et al (n = 91)

Malignancy type	Preceding LCH diagnosis	Concurrent with LCH diagnosis	After LCH diagnosis
Solid tumor	25	8	6
Lymphoma	3	3	3
Leukemia	3	0	2
Total	31	11	11
Egeler et al^15^			
Solid tumor	3	11	16
Lymphoma	11	24	4
Leukemia	2	6	14
Total	16	41	34

LCH, Langerhans cell histiocytosis.

### 3.3 LCH‐only cohort

Of the 132 adult LCH cohort, there were 90 patients with no other malignancies, presenting with LCH‐only disease. Median age was 42 years (range 18‐87) with a median follow‐up of 3.7 years (0.04‐20.1; Table 2). Overall survival at 3 years was 98% (Figure 2), which is notably higher when compared with the 82% in the additional malignancies cohort. Eighty (89%) patients were alive at last follow‐up (*P* = 0.008). Forty‐nine (54%) were smokers (*P* = 0.033), with an average of 33.1 pack‐years (2‐80). Sixty‐nine (77%) had single‐system disease (*P* = 0.038). Most common sites of LCH involvement were bone (48 patients; 53%), lung (15 patients; 17%), skin (21 patients; 23%), brain (14 patients; 16%), and liver (three patients; 3%). Less common sites of involvement were spleen, thyroid, and parotid, with one patient each.

### 3.4 Group 1: LCH and solid malignancies

There were 34 patients with 39 solid malignancies (Table 4) with a median age at diagnosis of 54 years (range 33‐89). Of the 34 patients, 20 were female (59%), 32 patients (94%) had single‐system disease, and 25 were smokers (74%). Sites of LCH most commonly included the lung (25 patients; 74%), bone (four patients; 12%), brain (two patients; 6%), and lymph nodes (two patients; 6%). Of the 34 solid malignancies, the most common solid tumor histologies were lung (18%), breast (18%), and colorectal cancer (12%; Table 4). Of the six patients with lung cancer, five were smokers (83%). Twenty‐one patients (62%) are alive. There were 16 patients presenting with solid tumors preceding LCH diagnosis for 25 malignancies, with a median time preceding LCH diagnosis of 8.4 years (range 0.4‐49.6). Eight patients presented with LCH concurrent with a solid tumor. Six patients developed a secondary solid tumor for six malignancies following LCH diagnosis with an average of 5.0 years after LCH diagnosis (range 0.27‐10.8).

**Table 4:** Additional malignancies by Langerhans cell histiocytosis group

Cancer	Number
Group 1: Solid malignancies	
Lung	6
Breast	6
Colorectal	4
Renal	3
Prostate	3
Melanoma	2
Germ cell tumor	2
Ovarian	1
Gastric	1
Liposarcoma	1
Cervical	1
Brain	1
Rhabdomyosarcoma	1
Leiomyosarcoma	1
Bladder	1
Intramedullary disease	1
Group 2: Lymphomas	
Follicular lymphoma	3
Hodgkin's lymphoma	3
Primary cutaneous B‐cell lymphoma	1
Primary cutaneous T‐cell lymphoma	1
Diffuse large B‐cell lymphoma	1
Lymphoma—not otherwise specified	1
Group 3: Other hematologic malignancies	
Acute myeloid leukemia	2
Multiple myeloma	2
Plasmacytoma	1
Myelodysplastic syndrome	1

### 3.5 Group 2: LCH and lymphomas

There were eight patients with a total of nine lymphomas, with a median age at diagnosis of 48 years (range 28‐65). Of the eight patients, six were females (67%), seven patients had single‐system disease (78%), and four were smokers (50%). The most common histologies were follicular and Hodgkin's lymphomas, with three patients each, and most common sites of LCH involvement included lymph nodes (four patients), skin (three patients), and lung (two patients). Six patients are alive. There were three patients presenting with lymphomas prior to LCH diagnosis, with a median time preceding LCH diagnosis of 5.0 years (2.9‐6.0). Three patients presented with a concurrent LCH and lymphoma diagnosis, and three patients developed lymphoma on average 3.7 years following their LCH diagnosis (range 0.75‐8.8).

### 3.6 Group 3: LCH and other hematologic malignancies

There were three patients with five hematologic malignancies, with a median age at diagnosis of 58 years (range 36‐70). Of the three patients, two were males (67%), two patients (67%) had single‐system disease, and two were smokers (67%). LCH involved the lung, skin, mucosa, and bone, and all three are alive. The most common hematologic malignancy was acute myeloid leukemia (two patients). Two patients presented with hematologic malignancies prior to LCH diagnosis, with a median time preceding LCH diagnosis of 9.7 years (range 9.5‐10.0).

## 4 DISCUSSION

Our cohort of adult LCH patients demonstrates an exceptionally high number of malignancies, particularly those preceding LCH diagnosis. This finding was particularly notable in the younger age cohorts (Table 1). Given the absence of a national database of LCH patients for comparison, the American Cancer Society report of cancer incidence in the United States in 2017 was used. The analysis is limited by the lack of a comparable matched cohort and the bias toward increased cancer diagnosis for patients with a prior oncologic diagnosis. Nonetheless, the notable increase in incidence among our cohort, particularly in the younger age groups, strongly supports an increased incidence of malignancy among LCH patients as compared to the overall population.

Solid tumors (74%) were the most common malignancy category, with lung and breast cancers comprising16% each of all solid tumors observed. Interestingly, the largest percentage of other malignancies was diagnosed prior to LCH diagnosis (Table 3); 58% of the 53 malignancies were diagnosed five or more years prior to LCH diagnosis. Our findings may suggest that in cancer patients, there is a subpopulation at increased susceptibility for development of LCH and additional malignancies. In this cohort, the biological etiology of LCH may precede cancer development by many years. The recent discovery of the prevalence of BRAF/ERK pathway mutations in LCH, observed in over 50% of cases, may be the causative factor for the increased rate of additional hematologic malignancies.^10^ The majority of LCH patients have either a *BRAF V600E* or *MAP2K1* mutation, and it has been theorized that the remainder may have other, currently unidentified mutations within the same pathway.^11^ Therefore, it is possible that an even larger percentage of LCH patients have a BRAF/ERK pathway alteration, predisposing them to hematologic malignancies through its critical role in cell cycle regulation and proliferation.

The prevalence of additional malignancies diagnosed prior to LCH is in contrast with results from the largest prior series of LCH patients with additional malignancies by Egeler et al,^14^ which included four patients from the authors’ institution, with an additional survey‐based compilation of 87 patients from 44 published case series or single case reports, which was published in 1993. This key study is the only prior large‐scale study of additional malignancies in LCH patients. Of the 87 patients, there were 53 adults and seven patients of unknown age where the majority of patients were diagnosed with an additional malignancy either concurrent with or after LCH diagnosis (Table 3). The temporal pattern was attributed by the authors to be treatment‐related due to the use of tumorigenic agents such as etoposide, which was widely used in the treatment of LCH during this time. Interestingly, the majority of lymphomas and leukemias were seen in pediatric patients, suggesting that the prevalence of solid malignancies in our cohort is an adult LCH‐specific profile. Alternatively, the underlying cause of tumorigenesis in the pediatric population may be the result of cytotoxic therapy or another independent pathway.

Etoposide is no longer commonly used in the treatment of LCH and notably, none of the patients in our cohort received etoposide for treatment of their LCH. This may allow us to examine the underlying biological processes leading to increased tumorigenesis more clearly without concern for potential confounding effects of treatment‐related sequelae. While solid tumors were the most common tumor type observed in our cohort (74%), the predominant type in the prior study was lymphoma, comprising 43% of the cohort. Of the 39 lymphomas observed in their cohort, 24 were diagnosed concurrently. This suggests the association between LCH and malignancy may represent a reactive process, which has been previously suggested for lymphomas.^19^

Comparison of the LCH cohort with additional malignancies and the LCH‐only cohort reveals several notable differences including a higher number of risk organ involvement in the LCH‐only group (20% vs 5%), fewer smokers (54% of the LCH‐only cohort vs 71%) with a lower average pack‐year (33 vs 35), and fewer deceased at last follow‐up (11% of the LCH‐only cohort vs 24%). Smoking history may have contributed to the number of additional malignancies. Overall survival at 3 years was 98% in the LCH‐only group compared with 82% in the additional malignancy cohort. The higher mortality seen in the additional malignancies group is expected and demonstrates that the second malignancies were the primary cause of morbidity rather than the LCH. The most common site of disease in the LCH‐only group was bone (53%), whereas lung was the most common site (60%) in the additional malignancy cohort. The majority of patients with lung LCH did not have documented pre‐existing lung pathology.

The predominant solid tumor observed in our cohort was lung cancer (six patients). Pulmonary LCH is more common in adults, although there is currently no known effective treatment other than smoking cessation. The Egeler et al study found that of patients diagnosed with both LCH and lung cancer, 75% were diagnosed concurrently. However, the presence of a local histiocytic reaction to lung cancer cannot be differentiated from a true diagnosis of LCH without additional histologic markers. Therefore, patients with a concurrent finding of lung cancer and pulmonary LCH were excluded from our study. The high rates of concurrent lung carcinoma and LCH diagnosis suggest that the association between LCH and malignancy may represent a specific dendritic cell reaction to the lymphoma or lung tumor. This also supports the prior conclusions from Egeler et al that the high number of combined cases may be due to a localized reactive process. Pulmonary LCH is thought to be associated with a separate disease process, and smoking cessation is the only known effective treatment.

Interestingly, lung LCH was the most common site of LCH involvement for patients with additional malignancies (60%) compared to 17% in the LCH‐only cohort. Although pack‐years of lung cancer patients were not reported in the prior study, our cohort of lung cancer and LCH patients had a mean of 34 pack‐years (range 0.6‐183; *P* = 0.033). This finding may be in part attributable to the increased diagnostic and staging workup of suspected lung cancer. Taken together with the higher rates of smoking and higher median pack‐years of the additional malignancy cohort compared to the LCH‐only cohort, our findings suggest environmental exposures such as cigarette smoke may cause a localized reactive process which contributes to additional tumorigenesis similar to the association of lung cancer with smoking. Nonetheless, the increased incidence of secondary malignancies has been previously documented in the literature, including a recent report noting the increased incidence of AML as a second primary malignancy in adult LCH by Goyal et al^20^ and a review of 102 patients with pulmonary LCH which noted a possible association with increased susceptibility to cancer development.^21^

LCH has previously been associated with increased cytokines, growth factors, and regulatory T‐cell expansion, suggesting a systemic inflammatory response.^10^ This inflammatory response may be associated with both the LCH and the concurrent lung cancer. Although previous literature has suggested that LCH may be a reactive process in response to existing lung cancer, the majority of patients in our cohort were diagnosed with a malignancy prior to their LCH diagnosis and did not have other known lung pathology. Of our 42‐patient cohort with 53 non‐LCH malignancies, 58% developed a non‐LCH cancer before LCH diagnosis. Therefore, we theorize that the association of LCH and additional cancers is due to a systemic inflammatory process, perhaps mild and chronic, which precedes both diseases. This is likely associated with smoking in the patients with lung cancer, although the etiology is unclear for the remaining non‐lung cases.

The high rates of additional malignancy in this single‐institutional cohort suggest that there is an association between LCH and additional tumorigenesis. Although our retrospective study is open to referral bias, our findings are consistent with numerous published small retrospective studies and case reports. The only other large‐scale study examining this research question included four patients from the authors’ institution along with a survey‐based compilation of an additional 87 patients, of which 61% were adults.^14^ It is unclear whether all patients met the histopathologic criteria for LCH diagnosis as outlined by the Histiocyte Society, due to the survey‐based method of compilation and with many cases occurring before 1987. The survey was distributed to authors who had previously published individual or small cohort case series observing the relationship between LCH and other malignancies, and therefore is open to recall bias and responder bias. The majority of patients in the prior series by Egeler et al were diagnosed with malignancies concurrently or after LCH diagnosis, rather than before LCH diagnosis. We note that the study was performed when etoposide was widely used; the authors therefore concluded that the association with malignancy was attributed to a localized reactive process or treatment with tumorigenic agents such as etoposide, which is no longer widely used for treatment of LCH. Importantly, the study established a possible link between LCH and additional malignancies, particularly in the pediatric population.

In our study, we observed an increased prevalence of other malignancies before or concurrent with LCH diagnosis. This observation suggests an additional cause of malignancy independent of modern LCH treatment in contrast to the previous era of etoposide use which the Egeler study reported on. In the context of the increased cytokines and growth factors long known to be associated with LCH, this may indicate a systemic biological process which precedes both LCH and other neoplastic processes, including potential contribution from random replicative errors.^22^ Our findings in this adult population demonstrate a tumorigenic profile distinct from the more commonly reported findings in the pediatric LCH population, suggesting separate tumorigenic profiles based on age.

In conclusion, to our knowledge, this is the first large‐scale single‐institution study examining additional malignancies in adult LCH patients, addressing an important gap in the literature. The association between additional malignancies and adult LCH appear concordant with the previous observations in the LCH population. Interestingly, the prevalence of solid tumors in our cohort suggests a distinct profile of additional malignancies specific to adult LCH, rather than the prevalence of leukemias and lymphomas seen in the pediatric LCH population. Mutations within the BRAF/ERK may be responsible for the increased tumorigenesis of these patients. Further exploration of the biology of this rare disease and its underlying processes is warranted to elucidate the mechanisms of increased association of malignancies in both adult and pediatric LCH patients.

## CONFLICT OF INTEREST

The authors have no conflict of interest to disclose.
